# Cut-off value for exercise-induced bronchoconstriction based on the features of the airway obstruction

**DOI:** 10.1371/journal.pone.0268969

**Published:** 2022-05-26

**Authors:** Noeul Kang, Eunsil Koh, Jin-Young Lee, Woo-Jung Song, Dong-Chull Choi, Byung-Jae Lee

**Affiliations:** 1 Division of Allergy, Department of Medicine, Samsung Medical Center, Sungkyunkwan University School of Medicine, Seoul, Republic of Korea; 2 Health Promotion Center, Samsung Medical Center, Seoul, Republic of Korea; 3 Division of Allergy and Clinical Immunology, Asan Medical Center, University of Ulsan College of Medicine, Seoul, Republic of Korea; National and Kapodistrian University of Athens, GREECE

## Abstract

The current cut-off value for diagnosing exercise-induced bronchoconstriction (EIB) in adults—percent fall in FEV_1_ (ΔFEV_1_) ≥ 10% after exercise challenge test (ECT)—has low specificity and weak evidences. Therefore, this study aimed to identify the cut-off value for EIB that provides the highest diagnostic sensitivity and specificity. Participants who underwent the ECT between 2007 and 2018 were categorized according to ΔFEV_1_: definite EIB (ΔFEV_1_ ≥ 15%), borderline (10% ≤ ΔFEV_1_ < 15%), and normal (ΔFEV_1_ < 10%). Distinct characteristics of the definite EIB group were identified and explored in the borderline EIB group. A receiver operating characteristic curve was plotted to determine the optimal cut-off value. Of 128 patients, 60 were grouped as the definite EIB group, 23 as the borderline group, and 45 as the normal group. All participants were men, with a median age of 20 years (interquartile range [IQR:] 19–23 years). The definite EIB group exhibited wheezing on auscultation (*P* < 0.001), ΔFEV_1_/FVC ≥ 10% (*P* < 0.001), and ΔFEF_25–75%_ ≥ 25% (*P* < 0.001) compared to other groups. Eight (8/23, 34.8%) patients in the borderline group had at least one of these features, but the trend was more similar to that of the normal group than the definite EIB group. A cut-off value of ΔFEV_1_ ≥ 13.5% had a sensitivity of 98.5% and specificity of 93.5% for EIB. Wheezing on auscultation, ΔFEV_1_/FVC ≥ 10%, and ΔFEF_25–75%_ ≥ 25% after ECT may be useful for the diagnosis of EIB, particularly in individuals with a ΔFEV_1_ of 10–15%. For EIB, a higher cut-off value, possibly ΔFEV_1_ ≥ 13.5%, should be considered as the diagnostic criterion.

## Introduction

Exercise-induced bronchoconstriction (EIB) is a transient narrowing of the lower airway during or after exercise [1–3]. Dyspnea and cough during physical activity are the classic symptoms of EIB; however, they have low sensitivity and specificity for predicting EIB [4–6]. EIB is diagnosed when lung function declines after an exercise challenge test (ECT). The difference between the lowest FEV_1_ value pre- and post-exercise, given as a percentage of the pre-exercise value obtained within 30 min after activity, is referred to as a percent fall in FEV_1_ (ΔFEV_1_) [4, 7]. The American Thoracic Society (ATS) suggests a post-exercise ΔFEV_1_ ≥ 10% to detect EIB, based on the results of ΔFEV_1_ in normal healthy participants without a family history of asthma, atopy, or recent upper respiratory tract infection [4]. However, supporting data comes from studies including children [8, 9] or a study involving both children and adults [10]. Although EIB is most commonly reported in schoolchildren, it also affects young adults, including athletes and military recruits [11, 12].

Compared with the recent guidelines, several other groups have suggested a ΔFEV_1_ ≥ 13% or even up to 15% for diagnosing EIB [13–16]. Furthermore, a positive challenge result of ΔFEV_1_ ≥ 20% is usually required in clinical trials to evaluate a drug for EIB [17]. These various criteria have resulted in a wide range of prevalence estimates for EIB and over-diagnosis of EIB [8, 18]. A lower cut-off value of ΔFEV_1_, such as suggested in the current guidelines, will increase the diagnostic sensitivity for EIB but at the expense of accuracy. Patients may be considered to have EIB even when they are clinically unaffected and do not require therapy. A precise diagnosis of EIB is required to identify acceptable levels of physical activity throughout life and reduce the potential impact of the disease on respiratory health. Therefore, in this study, we aimed to determine a cut-off value of ΔFEV_1_ with high diagnostic sensitivity and specificity for EIB by identifying and integrating the distinct features of airway obstruction.

## Material and methods

### Patients

This retrospective study was performed at Samsung Medical Center (a 1,997-bed tertiary referral hospital in Seoul, South Korea). Participants who underwent ECT due to current (< 1 month) experience of dyspnea on exertion between 2007 and 2018 were included and divided into three groups according to the ΔFEV_1_ value after the ECT: the definite EIB (ΔFEV_1_ ≥ 15%), borderline EIB (10% ≤ ΔFEV_1_ < 15%), and normal (ΔFEV_1_ < 10%) groups. Indicators of airway obstruction were identified in the definite EIB group by comparing with the other two groups, and these features were further investigated in the borderline EIB group. Data were retrieved from electronic medical records, including clinical variables and laboratory test results. The institutional review board of Samsung Medical Center approved this study (IRB no. 2019-03-041-002) and waived the requirement for informed consent owing to its retrospective nature.

### Exercise challenge test and measurements

Under the supervision of allergists, the ECT was performed according to the ATS standards [4], using a motor-driven treadmill with adjustable speed and grade in a dry air-conditioned room at 20 °C to 25 °C (< 15% relative humidity) at the specialized center for allergy. On the day of ECT, all patients were first assessed by the allergists before the challenge for any respiratory symptoms, and those with normal lung sounds on auscultation underwent ECT. After the ECT, localized lung sounds were not considered wheezing as they could also indicate central airway obstruction. The participants were instructed not to perform any rigorous physical activity or use short-acting β_2_-agonists for 24 hours before ECT. Spirometry was measured using a Vmax 22 instrument (SensorMedics, Yorba Linda, CA, USA) at baseline and after ECT (5, 10, 15, and 30 min after exercise), according to ATS/European Respiratory Society standards [19]. Absolute values were obtained, with the percent predicted (%pred) values of forced vital capacity (FVC), FEV_1_, FEV_1_/FVC, and FEF_25–75%_) calculated using data obtained from a representative Korean sample [20]. The best value with an appropriately performed flow-volume curve was chosen for the analysis.

To assess bronchial hyperresponsiveness (BHR) independently, a methacholine provocation test was performed on a day other than the day of the ECT [21, 22]. A positive test was defined as a concentration of methacholine less than 16 mg/mL that caused a 20% decrease in FEV1 (provocative concentration 20, PC20). PC_20_ levels between 4.0 and 16 mg/mL were considered borderline BHR, PC_20_ levels between 1.0 and 4.0 mg/mL were considered mild BHR, and PC_20_ levels below 1.0 mg/mL were considered moderate to severe BHR. The induced sputum, fraction of exhaled nitric oxide (FeNO), and skin prick tests were performed at the discretion of the attending allergist [23]. FeNO was measured using an NO analyzer (NIOX MINO; Aerocrine AB, Solna, Sweden) or NObreath (Bedfont Scientific, Maidstone, UK), according to the ATS guidelines.

### Statistical analysis

The categorical variables were presented as numbers (percentages), and the continuous variables were presented as median (interquartile range [IQR]). The categorical variables were compared using the Pearson x^2^ test or Fisher’s exact test, and the Kruskal–Wallis test (the non-parametric equivalent of one-way analysis of variance [ANOVA]) was used to compare the differences among the groups for continuous variables. *P-*values for pairwise group comparisons were obtained using a *post-hoc* Bonferroni test. Receiver operating characteristic (ROC) curves were plotted to obtain the optimal cut-off values of ΔFEV_1_ in determining EIB that yielded maximal sensitivity plus specificity. Statistical significance was defined as a two-sided *P*-value of < 0.05. All statistical analyses were performed using Statistical Analysis System (SAS) (version 9.4; SAS Institute, Inc., Cary, NC, USA) and R software (version 3.5.1; R Development Core Team, Vienna, Austria).

## Results

### Baseline characteristics

**Fig 1:**
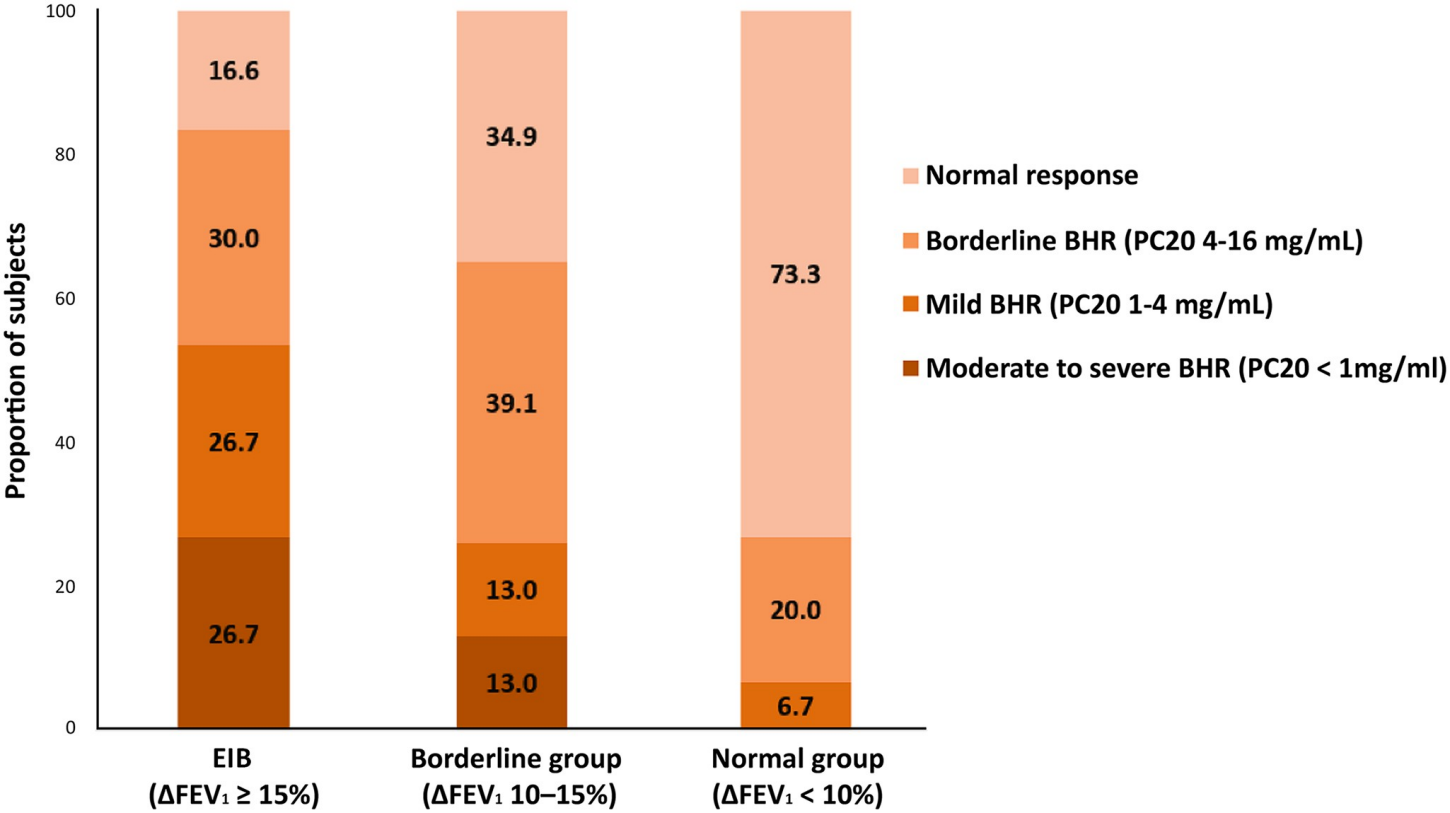
Proportion of patients according to the methacholine provocation test results.

Baseline characteristics of the 128 patients included in this study are shown in Table 1. The definite EIB group included 60 patients, borderline EIB group included 23, and normal group included 45. All patients were men, with a median age of 20 years (IQR: 19–23 years). Of 128 patients, 90 (70.3%) were never-smokers, while 38 (29.7%) had a smoking history; 33 (25.8%) were current smokers and 5 (3.9%) were ex-smokers. Concurrent asthma was identified in 59 (98.3%) of the EIB group compared with 16 (69.6%) in the borderline group and 16 (35.6%) in the normal group (*P* < 0.001). The definite EIB group had the lowest baseline FEV_1_ value of 92% (*P* = 0.038) and FEV_1_/FVC value of 81% (*P* < 0.001). The FVC values were not statistically different between the groups. FEF_25-75%_ values were different between the groups, both in L and %pred values (*P* = 0.001 for FEF_25-75%_, L/s, and *P* < 0.001 for FEF_25-75%_, %pred, respectively). The definite EIB group had the lowest FEF_25-75%_ value compared with those in the borderline EIB or normal group. There was more patients in the definite EIB group with FEF_25–75%_ <80% or FEF_25–75%_ <60% than borderline or normal group, but without statistical significance (*P* = 0.178 for FEF_25-75%_ < 80% and *P* = 0.311 for FEF_25-75%_ < 60%, respectively). Positive methacholine provocation test results were common in the definite EIB group (81.7% for the definite EIB, 69.6% for the borderline EIB, and 26.7% for the normal, *P* < 0.001). The definite EIB group had a higher proportion of moderate-to-severe BHR than the other groups (*P* < 0.001) (Fig 1).

**Table 1:** Baseline characteristics of the study population.

	Normal^a^ (n = 45)	Borderline^a^ (n = 23)	Definite EIB^a^ (n = 60)	*P*-value
Age, years	20 (19–23)	19 (18–21)	20 (19–22)	0.426
BMI (kg/m^2^)	23.4 (20.9–25.0)	21.8 (20.1–25.6)	24.3 (21.4–27.7)	0.085
Smoking history				**<0.001** ^c^ ^,^ ^d^
Never smoker	41 (91.1)	21 (91.3)	28 (46.7)	
Ever-smoked	4 (8.9)	2 (8.7)	32 (53.3)	
Concurrent asthma	16 (35.6)	16 (69.6)	59 (98.3)	**<0.001** ^b^ ^,^ ^c^ ^,^ ^d^
Baseline spirometry results				
FEV_1_, L	4.00 (3.62–4.26)	3.86 (3.53–4.20)	3.79 (3.48–4.23)	0.375
FEV_1_, %pred	97 (90–108)	98 (91–108)	92 (85–102)	**0.038** ^d^
FVC, L	4.60 (4.31–5.00)	4.71 (4.02–5.05)	4.73 (4.41–5.15)	0.397
FVC, %pred	94 (87–99)	91 (81–102)	95 (89–101)	0.480
FEV_1_/FVC	86 (79–91)	85 (79–93)	81 (75–85)	**<0.001** ^d^
FEF_25–75%_, L/s	4.28 (3.29–5.17)	4.37 (3.19–4.91)	3.58 (3.10–4.12)	**0.003** ^d^
FEF_25–75%_, %pred	96 (74–114)	89 (71–104)	80 (66–86)	**<0.001** ^d^
Methacholine provocation test	12 (26.7)	16 (69.6)	49 (81.7)	**<0.001** ^b^ ^,^ ^d^
PC20 < 1 mg/ml	0 (0.0)	3 (13.0)	16 (26.7)	
PC20 1–4 mg/ml	3 (6.7)	3 (13.0)	16 (26.7)	
PC20 4–16 mg/ml	9 (20.0)	9 (39.1)	18 (30.0)	
Laboratory results				
Skin prick test (+) (n = 116)	30 (79.0)	20 (87.0)	54 (98.2)	**0.006** ^d^
Total immunoglobulin E (kU/L) (n = 95)	150 (91–451)	494 (250–865)	329 (229–653)	**0.011**
Blood eosinophils (/μL) (n = 115)	213 (119–278)	371 (208–556)	324 (249–550)	**<0.001**
Sputum eosinophils (%) (n = 72)	3.3 (0–5.0)	4.0 (1.3–6.7)	4.0 (1.7–15.7)	0.076
≥ 3%	12 (54.6)	9 (64.3)	23 (63.9)	0.750
FeNO (ppb) (n = 38)	62 (40–76)	65 (38–139)	70 (41–129)	0.734
Positive (≥ 50 ppb)	9 (64.3)	2 (50.0)	14 (70.0)	0.792

^a^Normal (ΔFEV1 < 10%), borderline EIB (10% ≤ ΔFEV1 < 15%), and definite EIB groups (ΔFEV1 ≥ 15%).

^b^*P* < 0.05 with Bonferroni correction between the normal and borderline groups.

^c^*P* < 0.05, with Bonferroni correction between the borderline and definite EIB groups.

^d^*P* < 0.05, with Bonferroni correction between the normal and definite EIB groups.

A skin prick test was performed on 116 patients, and the patients in the definite EIB group showed the highest positive test results (98.2%, *P* = 0.006). The normal group had the lowest peripheral blood eosinophil counts (213/μL, *P* = 0.001). Sputum eosinophils and FeNO levels were the lowest in the normal group among the three groups, but the difference was not statistically significant.

### Identification of the indicators of airway obstruction

**Fig 2:**
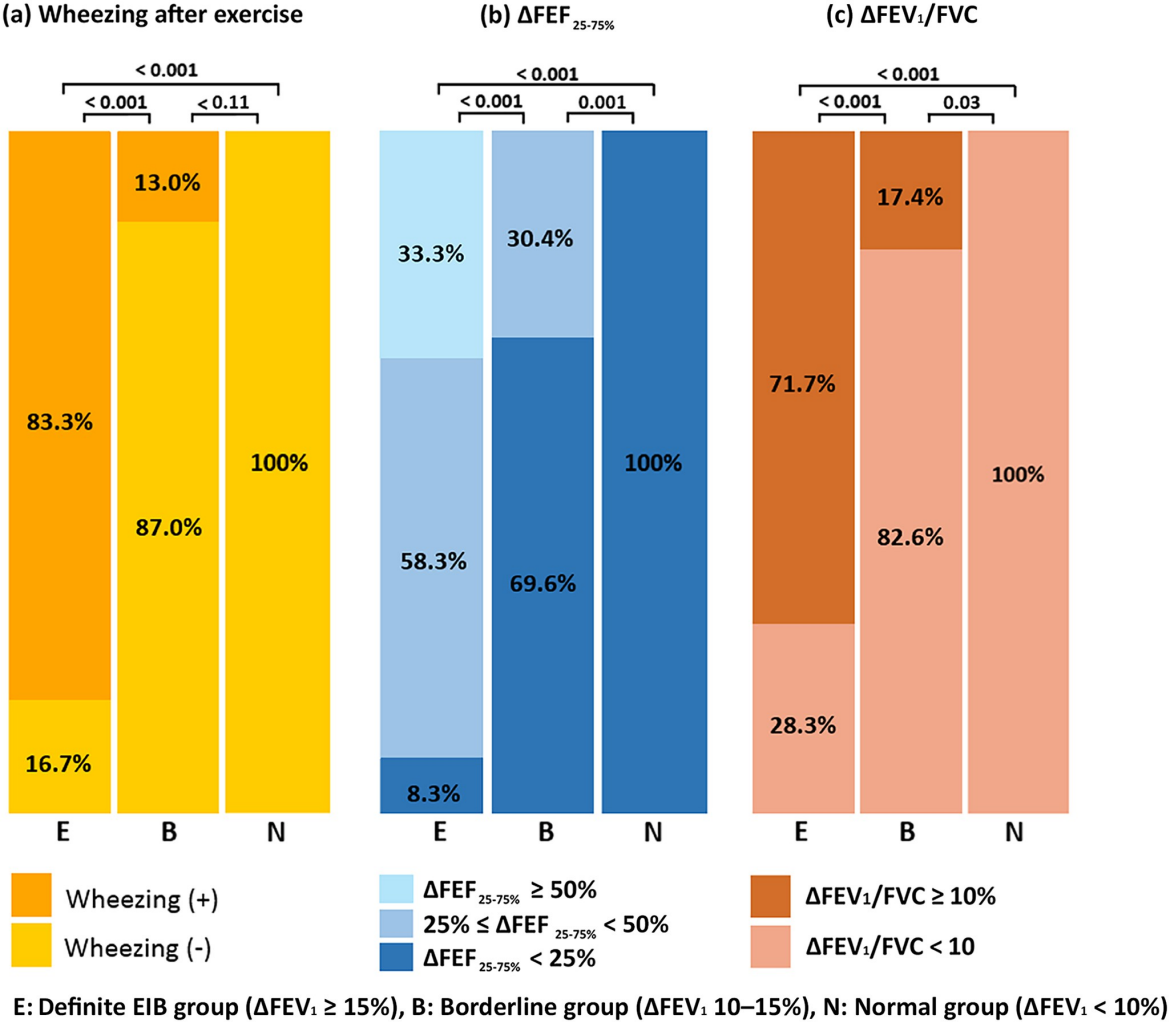
Distinct characteristics of the definite EIB group compared with the borderline EIB and normal groups.

Table 2 summarizes the changes in symptoms and pulmonary function test results after the ECT. Wheezing was auscultated in 50 (83.3%) patients in the definite EIB group, three (13.0%) in the borderline EIB group, and none in the normal group (*P* < 0.001). The definite EIB group (14.9%; IQR: 8.3–20.3%) had the greatest change in FEV_1_/FVC (ΔFEV_1_/FVC) before and after the ECT, followed by the borderline EIB group (3.2%; IQR: −1.5 to 8.7%) and the normal group (0.01%; IQR: −2.5 to 2.5%) (*P* < 0.001). In the definite EIB group, 43 (71.7%) patients had ΔFEV_1_/FVC of more than 10%. The median ΔFEF_25–75%_ was also higher in the definite EIB group (43.0%; IQR: 30.0–55.4%) than in the borderline EIB group (12.8%; IQR: 7.3–27.4%) or normal group (8.2%; IQR: 1.1–11.7%) (*P* < 0.001). In the definite EIB group, 20 (33.3%) had a ΔFEF_25–75%_ ≥ 50%, whereas all patients in the normal group had ΔFEF_25–75%_ less than ≤ 25%. Auscultated wheezing, ΔFEV_1_/FVC ≥ 10%, and ΔFEF_25–75%_ ≥ 25% after the ECT were distinct characteristics of the definite EIB group. Overall, the characteristics of the borderline EIB group were similar to those of the normal EIB group (Fig 2).

**Table 2:** Changes in symptoms and pulmonary function test results after ECT.

	Normal^a^ (n = 45)	Borderline^a^ (n = 23)	Definite EIB^a^ (n = 60)	*P*-value
Symptoms after ECT				
Wheezing	0 (0.0)	3 (13.0)	50 (83.3)	**<0.001**^c^^,^ ^d^
Cough	2 (4.4)	1 (4.3)	5 (8.3)	0.889
ΔFEV_1_/FVC (L)	0.01 (−2.5 to 2.5)	3.21 (−1.5 to 8.7)	14.9 (8.3–20.3)	**<0.001**^c^^,^ ^d^
ΔFEV_1_/FVC ≥ 10%	0 (0.0)	4 (17.4)	43 (71.7)	**<0.001**^c^^,^ ^d^
ΔFEF_25–75%_ (L/s)	8.2 (1.1–11.7)	12.8 (7.3–27.4)	43.0 (30.0–55.4)	**<0.001**^c^^,^ ^d^
0–25%	45 (100.0)	16 (69.6)	5 (8.3)	**<0.001**^c^^,^ ^d^
25–50%	0 (0.0)	7 (30.4)	35 (58.3)	
≥ 50%	0 (0.0)	0 (0.0)	20 (33.3)	
Time to lowest FEV_1_ (min)	10 (5–10)	7.5 (5–10)	10 (5–13)	0.601
0–5	10 (22.2)	2 (8.7)	2 (3.3)	**0.017**
5–10	10 (22.2)	10 (43.5)	24 (40.0)	
10–15	10 (22.2)	6 (26.1)	22 (36.7)	
≥ 15 or no decline	15 (33.3)	5 (21.7)	12 (20.0)	

^a^Normal (ΔFEV1 < 10%), borderline EIB (10% ≤ ΔFEV1 < 15%), and definite EIB groups (ΔFEV1 ≥ 15%).

^b^*P* < 0.05 with Bonferroni correction between the normal and borderline groups.

^c^*P* < 0.05, with Bonferroni correction between the borderline and definite EIB groups.

^d^*P* < 0.05, with Bonferroni correction between the normal and definite EIB groups.

### Optimal cut-off value for EIB

The distinct variables of the definite EIB group were further investigated in the borderline EIB group. In eight patients (8/23, 34.8%) in the borderline EIB group, at least one of these variables was identified, and all of them had a ΔFEF_25–75%_ ≥ 25%; moreover, six (75.0%) of them had either wheezing on auscultation or ΔFEV1/FVC ≥ 10% (Table 3).

**Table 3:** Indicators of airway obstruction in the borderline group (n = 23).

Patient no.	Baseline PFT		ΔFEV1(%)	Symptom after ECT		PFT after ECT	
	FEV_1_	FVC		Wheezing	Cough	ΔFEF_25–75%_ ≥ 25%	ΔFEV_1_/FVC ≥ 10%
**1**	4.52	5.46	14.7	-	-	**+**	**+**
**2**	2.47	3.34	14.6	**+**	+	**+**	-
**3**	3.53	4.45	14.5	-	-	**+**	**+**
**4**	3.84	4.21	14.4	-	-	**+**	**+**
5	3.25	4.79	14.2	-	-	**+**	-
**6**	4.10	4.41	13.9	-	-	**+**	-
**7**	4.79	5.05	13.6	**+**	-	**+**	-
8	3.84	4.03	13.6	-	-	-	-
9	3.75	3.9	13.6	-	-	-	-
10	3.59	3.84	12.9	-	-	-	-
**11**	3.86	5.22	12.7	**+**	-	**+**	**+**
12	3.88	4.02	11.6	-	-	-	-
13	4.07	4.71	11.5	-	-	-	-
14	4.15	4.71	10.9	-	-	-	-
15	3.50	3.94	10.9	-	-	-	-
16	4.11	4.42	10.8	-	-	-	-
17	3.81	5.02	10.8	-	-	-	-
18	4.89	5.84	10.8	-	-	-	-
19	4.63	5.79	10.6	-	-	-	-
20	4.20	4.94	10.5	-	-	-	-
21	2.87	3.51	10.2	-	-	-	-
22	3.30	4.72	10.0	-	-	-	-
23	4.28	5.16	10.0	-	-	-	-

The line divides the borderline group into two groups according to a 13.5% maximal fall in FEV_1_.

**Fig 3:**
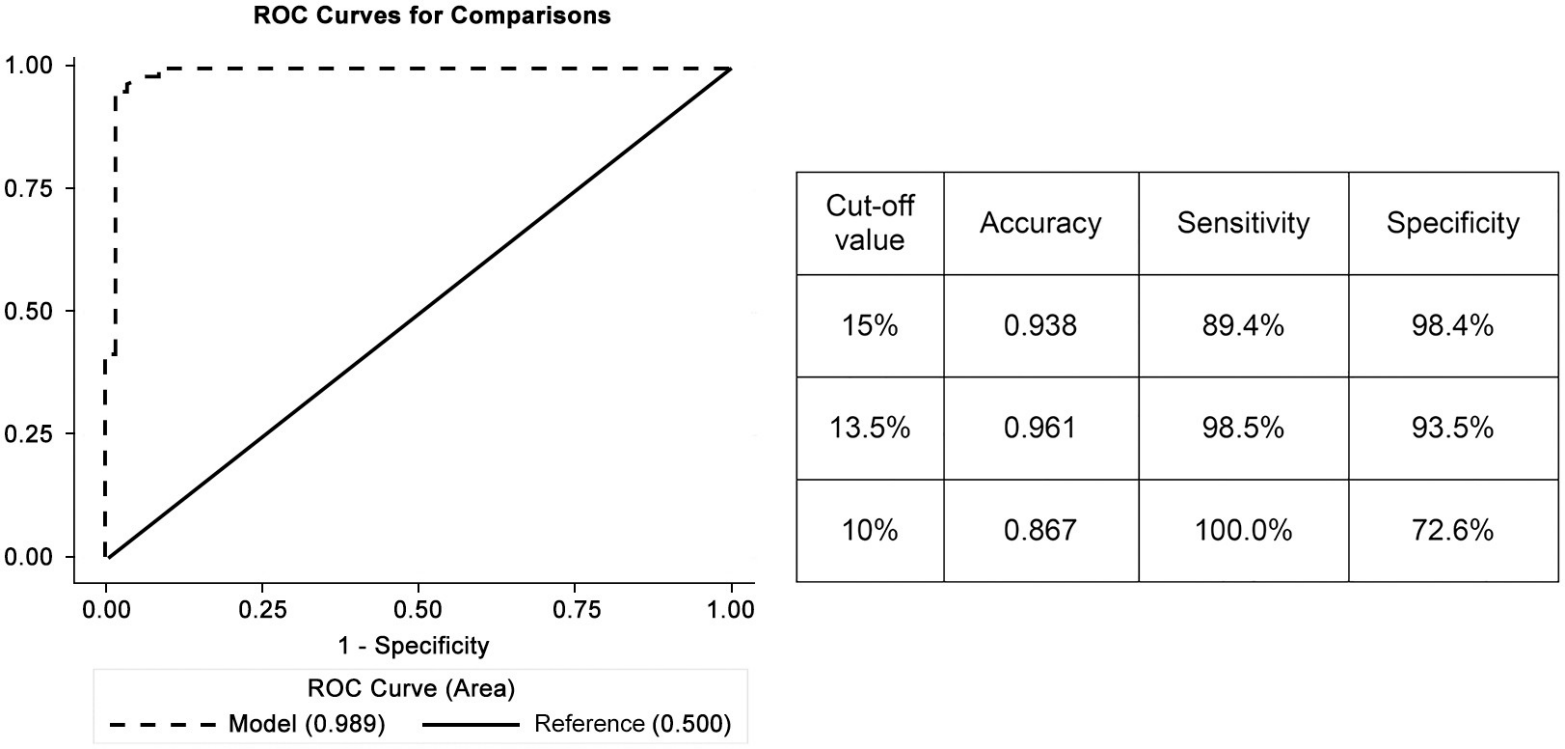
ROC curve, accuracy, sensitivity, and specificity of the cut-off values.

The ROC curve showed that the ΔFEV_1_ ≥ 10% as a cut-off value of EIB had an accuracy of 0.87 and 0.94 for ΔFEV_1_ ≥ 15% (AUC = 0.91; 95% CI: 0.85–0.96) (Fig 3). A ΔFEV_1_ cut-off value ≥ 13.5% had an accuracy of 96%, a sensitivity of 98.5%, and a specificity of 93.5%. In contrast, a ΔFEV_1_ ≥ 15% had a sensitivity and specificity of 89.4% and 98.4%, and a ΔFEV_1_ ≥ 10% had a sensitivity and specificity of 100% and 72.6%, respectively.

## Discussion

EIB occurs because of acute airway narrowing after exercise. In this study, the characteristics of airway obstruction were first identified in the definite EIB group (ΔFEV_1_ ≥ 15%), including wheezing on auscultation, ΔFEF_25–75%_ ≥ 25%, and ΔFEV_1_/FVC ≥ 10%. These three characteristics were not identified in the normal group, which is in line with results from earlier research [24–26]. Of the participants in the borderline EIB group with at least one of these characteristics, 87.5% had ΔFEV_1_ ≥ 13.6% and an estimated cut-off value of ΔFEV_1_ ≥ 13.5% showed high sensitivity (96.9%) and specificity (96.8%). A more significant and urgent treatment for EIB can be identified using this suggested cut-off.

With a cut-off value of ΔFEV_1_ ≥ 10%, the sensitivity was 100%, but the relatively low specificity would lead to a high false-positive rate. In light of the data from the previous studies, ΔFEV_1_ ≥ 15% after the ECT leaves no doubt in diagnosing EIB, whereas ΔFEV_1_ < 10% is commonly considered normal, which can exclude EIB. However, there was a gray zone of ΔFEV_1_ between 10–15%. Therefore, this group might be classified as either EIB or normal, depending on the arbitrary cut-off point used. We thoroughly evaluated the borderline EIB group in this study. Overall, the characteristics of the borderline EIB group were more similar to those of the normal group than to those of the definite EIB group (Fig 2). Among the patients in the borderline EIB group who showed at least one of the three characteristics of the definite EIB group, the ΔFEV_1_ value was more than 13.5% in most of them (7/8, 87.5%). Conversely, the distinct characteristics of the definite EIB group were not observed in most participants with ΔFEV_1_ < 13.6%, except for one patient (Patient No. 11 in Table 3). The patient had ΔFEV_1_ of 12.7% and exhibited all three features of airway obstruction.

Considering the symptoms after the ECT, wheezing on auscultation was notable in the definite EIB group, occurring in up to 80% patients, while coughing showed no significant statistical difference. Previous studies have reported that symptoms such as coughing or wheezing during sports had a lower diagnostic value. In this study, experienced allergists confirmed wheezing through close examination before and after the ECT, whereas in other studies self-reported wheezing was used [4–6].

ΔFEF_25–75%_ is another distinguishing trait of the definite EIB. FEF_25–75%_ assesses airway flow rates on an FVC segment and represents the initial changes associated with airflow obstruction in small airways [19, 27]. Therefore, it is more sensitive than FEV_1_ for evaluating EIB [24]. Currently, there is no recommendation on the utility of the percent predicted value of FEF_25-75%_, and in this manner, we measured the difference of FEF_25–75%_. Several studies have suggested a cut-off value of FEF_25-75%_ for evaluating small airway disease. Marseglia et al. suggested a cut-off < 80% [28], while Manoharan et al. suggested a stricter cut-off < 60% to define the presence of small airway disease [29]. In the present study, the borderline EIB group had a substantial decrease in FEF_25-75%_, even without symptoms. The definite EIB group had a higher proportion of ΔFEF_25–75%_ ≥ 25% than the borderline EIB or normal group. These findings are in line with prior research, which showed that a decrease in FEF_25–75%_ serves as an early signal of changes related to airflow obstruction in the small airways [30, 31].

The basal FEV_1_/FVC value was the lowest in the definite EIB group (definite EIB 81% *vs*. borderline 85% *vs*. normal 86%, *P* < 0.001) and showed the greatest difference before and after the ECT in the definite EIB group. More than 70% of the patients in the definite EIB group had a ΔFEV_1_/FVC > 10%, while this percentage was lower in the borderline EIB and normal groups (17.4% for the borderline EIB; 0% for the normal, respectively). The FEV_1_/FVC ratio has been used to express the degree of airway obstruction in children with asthma; however, its clinical implication in adults is unknown [32].

Atopic status is an important risk factor for the development of asthma and may contribute to the development of EIB. Atopic athletes are reported to have a higher risk of EIB than non-atopic athletes [33]. In a study by Koh *et al*., EIB-positive and-negative patients with asthma who underwent methacholine challenge and the degree of atopy between the two groups were compared [34]. The atopy score and skin reaction to house dust mites (*Dermatophagoides pteronyssinus*) significantly increased in patients with asthma and EIB compared with those without EIB, and the degree of EIB significantly correlated with the atopy score in all participants.

Regarding type 2 inflammation, FeNO and sputum eosinophilia were higher in the definite EIB group, although the difference was insignificant. FeNO and sputum eosinophilia were not useful in this population, but they suggest and support the finding that type 2 inflammation is not significant in mild EIB [35]. FeNO, a marker of type 2 inflammation in the bronchial mucosa, has a high predictive value for EIB in patients with asthma, but its relationship with this condition needs to be investigated further [36, 37].

This study had several limitations. First, the ECT was performed only once; two tests may be required when using exercise to exclude a diagnosis of EIB [4]. However, this suggestion is based on a criterion for cut-off ΔFEV1 ≥ 10%. Moreover, even when considering ΔFEV1 ≥ 10% as the cut-off, the reproducibility of EIB determined by two separate tests is high [10]. We also performed a methacholine provocation test on all participants. Indirect challenges are more specific in reflecting bronchial hyper-responsiveness, and direct challenges, such as methacholine, are not useful for detecting EIB because they have low sensitivity. However, the methacholine provocation test showed an excellent negative predictive value [38] and may have a supplementary role in excluding ECT, although this was not investigated in this study. Second, because this study was conducted at a single referral center with only young male patients, selection bias may restrict the generalizability of the major findings. All participants with dyspnea during or shortly after exercise were included in the study, regardless of whether they were athletes or with asthma. This study reflects the real-world.

In conclusion, the characteristics of airway obstruction, such as wheezing on auscultation, ΔFEV_1_/FVC ≥ 10%, and ΔFEF_25–75%_ ≥ 25% after ECT, may be useful for the diagnosis of EIB, particularly in individuals with a ΔFEV_1_ of 10–15%. For EIB, a higher cut-off value, possibly ΔFEV_1_ ≥ 13.5%, should be considered as the diagnostic criterion.

## References

[1] Weiler JM, Brannan JD, Randolph CC, Hallstrand TS, Parsons J, Silvers W, et al. Exercise-induced bronchoconstriction update-2016. J Allergy Clin Immunol. 2016; 138:1292–5 e36. doi: 10.1016/j.jaci.2016.05.029 27665489

[2] Beck KC, Offord KP, Scanlon PD. Bronchoconstriction occurring during exercise in asthmatic subjects. Am J Respir Crit Care Med. 1994; 149:352–7. doi: 10.1164/ajrccm.149.2.8306029 8306029

[3] McFadden ER Jr., Gilbert IA. Exercise-induced asthma. N Engl J Med. 1994; 330:1362–7. doi: 10.1056/NEJM199405123301907 8152449

[4] Parsons JP, Hallstrand TS, Mastronarde JG, Kaminsky DA, Rundell KW, Hull JH, et al. An official American Thoracic Society clinical practice guideline: exercise-induced bronchoconstriction. Am J Respir Crit Care Med. 2013; 187:1016–27. doi: 10.1164/rccm.201303-0437ST 23634861

[5] Parsons JP, Kaeding C, Phillips G, Jarjoura D, Wadley G, Mastronarde JG. Prevalence of exercise-induced bronchospasm in a cohort of varsity college athletes. Med Sci Sports Exerc. 2007; 39:1487–92. doi: 10.1249/mss.0b013e3180986e45 17805078

[6] Weiler JM, Bonini S, Coifman R, Craig T, Delgado L, Capao-Filipe M, et al. American Academy of Allergy, Asthma & Immunology Work Group report: exercise-induced asthma. J Allergy Clin Immunol. 2007; 119:1349–58. doi: 10.1016/j.jaci.2007.02.041 17433829

[7] Global Initiative for Asthma. Global Strategy for Asthma Management and Prevention, 2018. www.ginasthma.org. Accessed June 1, 2020.

[8] Custovic A, Arifhodzic N, Robinson A, Woodcock A. Exercise testing revisited. The response to exercise in normal and atopic children. Chest. 1994; 105:1127–32. doi: 10.1378/chest.105.4.1127 8162737

[9] Kattan M, Keens TG, Mellis CM, Levison H. The response to exercise in normal and asthmatic children. J Pediatr. 1978; 92:718–21. doi: 10.1016/s0022-3476(78)80135-8 641618

[10] Anderson SD, Pearlman DS, Rundell KW, Perry CP, Boushey H, Sorkness CA, et al. Reproducibility of the airway response to an exercise protocol standardized for intensity, duration, and inspired air conditions, in subjects with symptoms suggestive of asthma. Respir Res. 2010; 11:120. doi: 10.1186/1465-9921-11-120 20807446 PMC2939602

[11] Rundell KW, Im J, Mayers LB, Wilber RL, Szmedra L, Schmitz HR. Self-reported symptoms and exercise-induced asthma in the elite athlete. Med Sci Sports Exerc. 2001; 33:208–13. doi: 10.1097/00005768-200102000-00006 11224807

[12] Sinclair DG, Sims MM, Hoad NA, Winfield CR. Exercise-induced airway narrowing in army recruits with a history of childhood asthma. Eur Respir J. 1995; 8:1314–7. doi: 10.1183/09031936.95.08081314 7489796

[13] Cockcroft D, Davis B. Direct and indirect challenges in the clinical assessment of asthma. Ann Allergy Asthma Immunol. 2009; 103:363–9; quiz 9–72, 400. doi: 10.1016/S1081-1206(10)60353-5 19927533

[14] Crapo RO, Casaburi R, Coates AL, Enright PL, Hankinson JL, Irvin CG, et al. Guidelines for methacholine and exercise challenge testing-1999. This official statement of the American Thoracic Society was adopted by the ATS Board of Directors, July 1999. Am J Respir Crit Care Med. 2000; 161:309–29. doi: 10.1164/ajrccm.161.1.ats11-99 10619836

[15] Godfrey S, Springer C, Bar-Yishay E, Avital A. Cut-off points defining normal and asthmatic bronchial reactivity to exercise and inhalation challenges in children and young adults. Eur Respir J. 1999; 14:659–68. doi: 10.1034/j.1399-3003.1999.14c28.x 10543290

[16] Rundell KW, Slee JB. Exercise and other indirect challenges to demonstrate asthma or exercise-induced bronchoconstriction in athletes. J Allergy Clin Immunol. 2008; 122:238–46; quiz 47–8. doi: 10.1016/j.jaci.2008.06.014 18678339

[17] Industry. Gf. Exercise-induced Bronchospasm (EIB) development of Drugs to Prevent EIB. In: U.S. Department of Health and Human Services FaDA, Centre for Drug Evaluation and Research, editor. 2002.

[18] Weiler JM, Hallstrand TS, Parsons JP, Randolph C, Silvers WS, Storms WW, et al. Improving screening and diagnosis of exercise-induced bronchoconstriction: a call to action. J Allergy Clin Immunol Pract. 2014; 2:275–80 e7. doi: 10.1016/j.jaip.2013.11.001 24811017

[19] Miller MR, Hankinson J, Brusasco V, Burgos F, Casaburi R, Coates A, et al. Standardisation of spirometry. Eur Respir J. 2005; 26:319–38. doi: 10.1183/09031936.05.00034805 16055882

[20] Choi Jung Keun, Paek Domyung, Oh Lee Jeoung. Normal predictive values of spirometry in Korean population. Tuberculosis and Respiratory Diseases. 2005; 58:230–42. doi: 10.4046/trd.2005.58.3.230

[21] Fitch KD, Sue-Chu M, Anderson SD, Boulet LP, Hancox RJ, McKenzie DC, et al. Asthma and the elite athlete: summary of the International Olympic Committee’s consensus conference, Lausanne, Switzerland, January 22–24, 2008. J Allergy Clin Immunol. 2008; 122:254–60, 60 e1–7. doi: 10.1016/j.jaci.2008.07.003 18678340

[22] Cockcroft DW. Bronchoprovocation methods: direct challenges. Clin Rev Allergy Immunol. 2003; 24:19–26. doi: 10.1385/CRIAI:24:1:19 12644716

[23] Dweik RA, Boggs PB, Erzurum SC, Irvin CG, Leigh MW, Lundberg JO, et al. An official ATS clinical practice guideline: interpretation of exhaled nitric oxide levels (FENO) for clinical applications. Am J Respir Crit Care Med. 2011; 184:602–15. doi: 10.1164/rccm.9120-11ST 21885636 PMC4408724

[24] Fonseca-Guedes CH, Cabral AL, Martins MA. Exercise-induced bronchospasm in children: comparison of FEV1 and FEF25-75% responses. Pediatr Pulmonol. 2003; 36:49–54. doi: 10.1002/ppul.10309 12772223

[25] Marini JJ, Pierson DJ, Hudson LD, Lakshminarayan S. The significance of wheezing in chronic airflow obstruction. Am Rev Respir Dis. 1979; 120:1069–72. doi: 10.1164/arrd.1979.120.5.1069 507523

[26] Shim CS, Williams MH Jr. Relationship of wheezing to the severity of obstruction in asthma. Arch Intern Med. 1983; 143:890–2. 6679232

[27] Pellegrino R, Viegi G, Brusasco V, Crapo RO, Burgos F, Casaburi R, et al. Interpretative strategies for lung function tests. Eur Respir J. 2005; 26:948–68. doi: 10.1183/09031936.05.00035205 16264058

[28] Marseglia GL, Cirillo I, Vizzaccaro A, Klersy C, Tosca MA, La Rosa M, et al. Role of forced expiratory flow at 25–75% as an early marker of small airways impairment in subjects with allergic rhinitis. Allergy Asthma Proc. 2007; 28:74–8. doi: 10.2500/aap.2007.28.2920 17390762

[29] Manoharan A, Anderson WJ, Lipworth J, Lipworth BJ. Assessment of spirometry and impulse oscillometry in relation to asthma control. Lung. 2015; 193:47–51. doi: 10.1007/s00408-014-9674-6 25516285

[30] Fuentes C, Contreras S, Padilla O, Castro-Rodriguez JA, Moya A, Caussade S. Exercise challenge test: is a 15% fall in FEV(1) sufficient for diagnosis? J Asthma. 2011; 48:729–35. doi: 10.3109/02770903.2011.594139 21749286

[31] Lipworth BJ, Clark DJ. Effects of airway calibre on lung delivery of nebulised salbutamol. Thorax. 1997; 52:1036–9. doi: 10.1136/thx.52.12.1036 9516895 PMC1758465

[32] Strunk RC, Weiss ST, Yates KP, Tonascia J, Zeiger RS, Szefler SJ. Mild to moderate asthma affects lung growth in children and adolescents. J Allergy Clin Immunol. 2006; 118:1040–7. doi: 10.1016/j.jaci.2006.07.053 17088127

[33] Sallaoui R, Chamari K, Mossa A, Tabka Z, Chtara M, Feki Y, et al. Exercise-induced bronchoconstriction and atopy in Tunisian athletes. BMC Pulm Med. 2009; 9:8. doi: 10.1186/1471-2466-9-8 19196480 PMC2661040

[34] Koh YI, Choi IS, Lim H. Atopy may be related to exercise-induced bronchospasm in asthma. Clin Exp Allergy. 2002; 32:532–6. doi: 10.1046/j.0954-7894.2002.01353.x 11972598

[35] Duong M, Subbarao P, Adelroth E, Obminski G, Strinich T, Inman M, et al. Sputum eosinophils and the response of exercise-induced bronchoconstriction to corticosteroid in asthma. Chest. 2008; 133:404–11. doi: 10.1378/chest.07-2048 18071011

[36] Anderson SD. Indirect challenge tests: Airway hyperresponsiveness in asthma: its measurement and clinical significance. Chest. 2010; 138:25S–30S. doi: 10.1378/chest.10-0116 20668015

[37] Buchvald F, Hermansen MN, Nielsen KG, Bisgaard H. Exhaled nitric oxide predicts exercise-induced bronchoconstriction in asthmatic school children. Chest. 2005; 128:1964–7. doi: 10.1378/chest.128.4.1964 16236842

[38] Holzer K, Anderson SD, Douglass J. Exercise in elite summer athletes: Challenges for diagnosis. J Allergy Clin Immunol. 2002; 110:374–80. doi: 10.1067/mai.2002.127784 12209082

